# Perihepatic Abscess due to a Liver Suture with Pledgets Used to Treat a Penetrating Liver Injury

**DOI:** 10.1155/2021/6817617

**Published:** 2021-10-06

**Authors:** Hazuki Koguchi, Kimihiko Kusashio, Akihiro Fujita, Nao Yamamoto

**Affiliations:** Department of Surgery, Chiba Rosai Hospital, 2-16, Tatsumidai Higashi, Ichihara-shi, Chiba-ken, Japan

## Abstract

**Background:**

Selective nonoperative management has become the standard for liver injuries. Accordingly, we cannot perform surgery for liver injuries as frequently as in the past. This report is aimed at sharing a valuable experience of postoperative complications after surgery for a liver injury. *Case Presentation*. A 40-year-old man was stabbed in his abdomen and underwent an emergency laparotomy for a severe liver injury. Five months after the operation, he developed fever, and purulent discharge was observed from an abdominal fistula. He was diagnosed with a perihepatic abscess and duodenal perforation due to the pledgets used for the operation. He underwent a second surgery to remove the pledgets and the abscess cavity for infection control and was discharged in good condition.

**Conclusion:**

The intra-abdominal environment should be considered contaminated due to bile leakage in surgeries following liver injury. Furthermore, nonabsorbable agents should not be used in these contaminated areas.

## 1. Introduction

Selective nonoperative management has become the standard of therapy for blunt liver injuries in this century [1]. Nonoperative management of penetrating liver injuries has also become more prevalent in recent years [2]. The number of liver injury cases that require surgical treatment may be decreasing; in addition, the surgical strategy is not standardized. Hence, technical tips and pitfalls of surgery for liver injuries are valuable for surgeons, especially acute care surgeons.

We present a case of a perihepatic abscess due to the use of pledgets for a penetrating liver injury.

## 2. Case Presentation

A 40-year-old man presented to the emergency department because of an abdominal stab wound.

**Figure 1:**
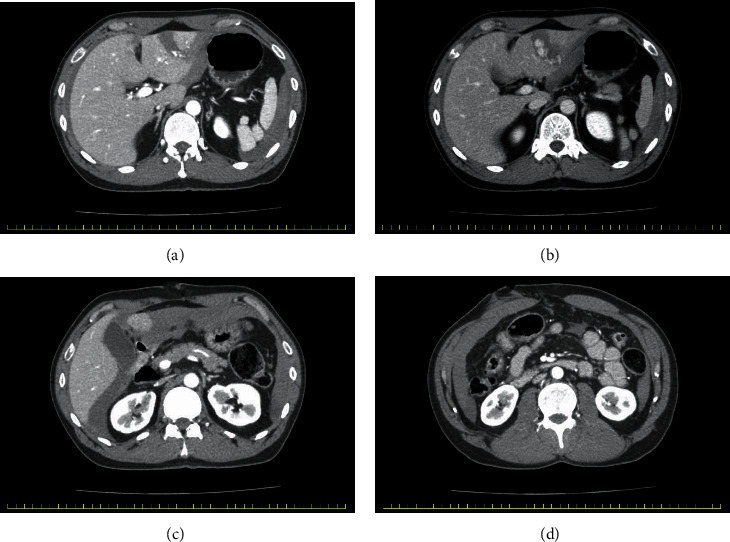
Contrast-enhanced computed tomography of the abdomen, taken before the initial operation, shows a laceration of the left lateral segment of the liver and extravasations (a, b), hematoma in the Morrison's pouch (c), and the penetration area (d).

He was diagnosed with a severe liver injury and underwent an emergency laparotomy (Figure 1).

This revealed a laceration in the left lateral segment of the liver (grade IV). Active bleeding was stopped by direct suturing of the exposed arteries and veins after perihepatic packing and by using the Pringle maneuver. Hepatorrhaphy using a thread (nonabsorbable monofilament) with a polytetrafluoroethylene (PTFE) pledget was performed on the laceration to the liver.

After the initial operation, the doctors recognized a leak of pancreatic juice from the abdominal drain. They assessed the pancreatic duct injuries with an endoscopic retrograde cholangiopancreatography. On postoperative day 4, he underwent distal pancreatectomy and splenectomy. The spleen could not be preserved because of inflammatory tissues around the pancreas.

After the management of a postoperative pancreatic fistula, the patient was discharged from the hospital with an abdominal drain.

**Figure 2:**
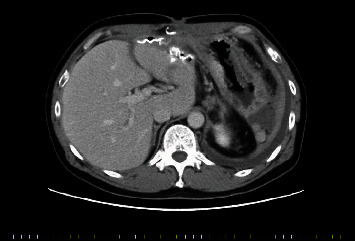
Abdominal computed tomography shows a perihepatic abscess around foreign bodies with air bubbles 5 months after the initial operation.

Two months after the initial operation, he was referred to our hospital and received follow-up care of the fistula as an outpatient. Five months after the initial operation, he was urgently hospitalized due to fever, purulent discharge from the fistula, and surgical wound dehiscence. Computed tomography (CT) showed a perihepatic abscess around the foreign bodies, which were suspected to be pledgets (Figure 2). Fistulography showed a path between the fistula and duodenum. The drain fluid culture was positive for *Streptococcus anginosus*. He was diagnosed with a perihepatic abscess and duodenal perforation associated with pledgets used for the initial operation. He underwent surgery for removal of the pledgets and abscess cavities for infection control.

### 2.1. Operative Findings

Pledgets were present in the abscess in segment III. The abscess adhered to the duodenal bulb, hepatoduodenal ligament, and gallbladder. The perforation site was exposed at the duodenal bulb when adhesions around the abscess were separated. The perforation was suspected to have been caused by inflammatory cell infiltration. Finally, we performed partial hepatectomy, cholecystectomy, and direct suture and omental implantation for the duodenal perforation. The patient was discharged from our hospital on postoperative day 15.

**Figure 3:**
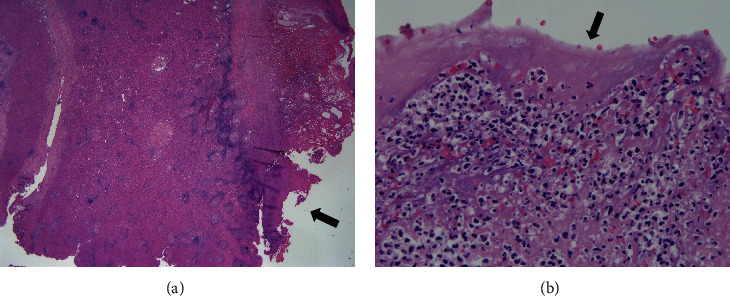
Pathological findings (hematoxylin and eosin stain, magnification: (a) 1x; (b) 40x). Fibrin deposition and neutrophil infiltration were found in the contact area of the pledgets.

Pathological findings showed fibrin deposition and neutrophil infiltration around the contact area of the pledgets. This reveals that the area was continuously infected (Figure 3).

## 3. Discussion

Recently, nonoperative management has been the main treatment for blunt liver injuries [1]. The adaptation of nonoperative management for penetrating liver injuries has also been considered in recent years [2, 3]. Demetriades et al. described that liver injury patients with hemodynamically unstable condition, peritonitis, or unevaluable abdomen due to brain or spinal cord injury or who need other operations under general anesthesia should undergo operative management [2].

The major ways to control bleeding from the liver are vessel ligation, hepatorrhaphy, manual compression, and partial resection. Hepatorrhaphy is only used to control bleeding in the liver parenchyma. Manual compression or partial resection is useful for a deep laceration of the liver. In this case, direct vessel ligation and hepatorrhaphy with pledgets were performed.

If a patient is hemodynamically stable and there is a team available with sufficient skill to perform a laparoscopic hepatic surgery, a laparoscopic approach would be worth considering as a minimally invasive surgery. There are some reports of laparoscopic lavage and liver resection in patients in whom nonoperative management failed because of biliary peritonitis [4, 5].

Liver injuries result in a contaminated environment caused by bile leakage at the laceration [6]. When this area is infected, it becomes a refractory abscess, especially if pledgets and foreign bodies are present.

The drain fluid culture in this case was positive for *Streptococcus anginosus*, which is part of the normal bacterial flora in the oral cavity and gastrointestinal tract. However, this does not contradict the fact that the abscess was caused by a persistent infection from bile leakage.

We found three case reports about liver abscesses after a liver suture with PTFE pledgets.

Kohama et al. reported a liver abscess diagnosed 27 months after a liver suture for blunt liver injury. Food residue flowed out of the fistula. Fistulography and a CT scan showed a liver abscess with pledget and duodenal perforation [7].

You et al. reported the case of a patient who complained of early satiety and weight loss 9 years after nonanatomic liver resection with PTFE pledgets for hepatocellular carcinoma. Esophagogastroduodenoscopy (EGD) revealed a pledget to the duodenal lumen with mucosal ulceration [8].

The last case involved a patient with recurrent liver abscesses 40 years after undergoing a partial hepatectomy. EGD showed a pledget through the fistula opening into the duodenal bulb, and fistulography revealed a tract into the liver [9].

For all three aforementioned cases, surgical or endoscopic removal of pledgets was required for complete infection control.

It is most important to control active bleeding and infection during surgery for severe abdominal injuries. However, we should also develop surgical strategies that decrease the possibility of postoperative complications requiring reoperation.

For instance, hepatorrhaphy with absorbable polyglycolic acid (PGA) pledgets would worth being considered for a severe liver injury. There are no reports on postoperative abscesses associated with PGA pledgets. The usefulness of PGA pledgets for a liver surgery is still a controversial topic. Further research is required to detect more effective materials for reinforcement and prevention of bile leakage and perihepatic abscess in surgery for severe liver injury.

In conclusion, the intra-abdominal environment should be considered contaminated due to bile leakage in surgeries following liver injury. Hepatorrhaphy with PTFE pledgets should not be performed for a liver injury because of the risk of refractory abscess that requires surgical treatment. Further research is required to identify more effective materials that combine reinforcement and prevention of bile leakage in surgery for severe liver injury.

## Data Availability

The data that support the findings of this study are available on request from the corresponding author, Hazuki Koguchi.
